# The metastatic niche formation: focus on extracellular vesicle-mediated dialogue between lung cancer cells and the microenvironment

**DOI:** 10.3389/fonc.2023.1116783

**Published:** 2023-05-03

**Authors:** Francesca Pontis, Luca Roz, Orazio Fortunato, Giulia Bertolini

**Affiliations:** Epigenomics and Biomarkers of Solid Tumors, Department of Experimental Oncology, Fondazione IRCCS Istituto Nazionale dei Tumori, Milan, Italy

**Keywords:** extracellular vesicles (EV), premetastatic niche, lung cancer, dormancy (seed), CTC (circulation tumor cells)

## Abstract

Lung cancer is the deadliest cancer in the world, with the majority of patients presenting with advanced or metastatic disease at first diagnosis. The lungs are also one of the most common sites of metastasis from lung cancer and other tumors. Understanding the mechanisms that regulate metastasis formation from primary lung cancer and in the lungs is therefore fundamental unmet clinical need. One of the first steps during the establishment of lung cancer metastases includes the formation of the pre-metastatic niche (PMN) at distant organs, which may occur even during the early phases of cancer development. The PMN is established through intricate cross-talk between primary tumor-secreted factors and stromal components at distant sites. Mechanisms controlling primary tumor escape and seeding of distant organs rely on specific properties of tumor cells but are also tightly regulated by interactions with stromal cells at the metastatic niche that finally dictate the success of metastasis establishment. Here, we summarize the mechanisms underlying pre-metastatic niche formation starting from how lung primary tumor cells modulate distant sites through the release of several factors, focusing on Extracellular Vesicles (EVs). In this context, we highlight the role of lung cancer-derived EVs in the modulation of tumor immune escape. Then, we illustrate the complexity of Circulating Tumor Cells (CTCs) that represent the seeds of metastasis and how interactions with stromal and immune cells can help their metastatic dissemination. Finally, we evaluate the contribution of EVs in dictating metastasis development at the PMN through stimulation of proliferation and control of disseminated tumor cell dormancy. Overall, we present an overview of different steps in the lung cancer metastatic cascade, focusing on the EV-mediated interactions between tumor cells and stromal/immune cells.

## 1 The lungs as source and target of metastases

Lung cancer is one of the most common cancers and is still the leading cause of cancer-related deaths. Indeed, as reported by GLOBOCAN, lung cancer is the second most prevalent neoplasm and the main cause of cancer mortality, with 2.2 million newly diagnosed cases and 1.8 million deaths in 2020. In most countries, the 5-year survival rate of lung cancer patients is only 10% to 20%: stratifying by disease stage, only 60% of stage IB, 50% of stage II, and 40% of stage IIIA patients survive more than 5 years (1). The lack of symptoms in the initial stages makes early diagnosis difficult, often delaying lung cancer detection at an advanced or widely metastatic stage (2).

Several efforts have been made to improve the therapeutic management of advanced lung cancer including specific target therapies against driver mutations and, more recently, the introduction of immunotherapies alone or in combination with standard chemotherapy. Immune-checkpoint inhibitors (ICI) have demonstrated efficacy in the treatment of advanced lung cancer even though a sizable proportion of patients, unfortunately, do not respond to treatment or have a durable response. Despite treatment improvement, the 5-year survival rate for advanced lung cancer remains very low overall (6%) (3).

The survival rate of lung cancer patients is strongly affected by the presence of advanced or metastatic disease which often occurs quickly or is already present at diagnosis. It has been estimated that almost 70% of the total lung cancer-related deaths are a consequence of metastatic spread, thus representing an extremely relevant clinical and social issue (1). Moreover, despite the common belief that metastatic dissemination might be related to late-stage disease, cancer cell dissemination could be an early event in cancer progression (4, 5).

One of the earliest events of the metastatic process is the escape of cancer cells from the primary tumor, often linked to the acquisition of mesenchymal and invasive properties through the induction of the epithelial-to-mesenchymal transition process (EMT), allowing cancer cells to intravasate into the lymphatic or blood circulatory systems. Then, cancer cells traveling in the circulation, alone or within a cluster of cells, can reach distant sites where they can extravasate and grow as metastatic lesions. All these processes require a complex network of communications among different cell types and tissues. In the attempt to fully elucidate the metastatic process and the crosstalk among different cellular players, experimental studies have often focused on cancer dissemination to the lungs since it represents one of the most frequent sites of metastasis from many tumor types, including carcinomas of the breast, colon, kidney, melanoma, and lung cancer itself (6). In fact, it must be emphasized that primary lung cancer also frequently metastasizes to the lung: indeed, lung cancer is the tumor that contributes most to the incidence rate of synchronous lung metastases (41%), followed by colon and rectum (10%), kidney (7%), pancreas (7%) and breast (6%) (7).

The preferential sites for lung cancer-derived metastasis at diagnosis are lung (49.0-59.2%), brain (29.9-41.9%), bone (28.5-38.8%), liver (13.2-26.3%), and adrenal gland (10.1-24.1%) (8). In detail, among the most frequent form of lung cancer, non-small cell lung cancer (NSCLC), different histological subtypes can preferentially metastasize to specific organs: squamous cell lung carcinoma (SCLC) often spreads to the liver whereas adenocarcinoma most frequently spreads to brain. Moreover, specific genetic alterations could influence the metastatic pattern as in the presence of EGFR mutations which have been linked to brain metastasis in NSCLC (9). These differences in metastatic tropism for lung cancer subtypes may also reflect different interactions between tumor cells and specific microenvironments (6).

This clinical evidence underlines the need to further elucidate the mechanisms behind lung cancer tropism to certain organs. Additionally, information gathered on the pulmonary ‘soil’ as permissive or restrictive for the growth of cancer cells of different origins may also provide important insights into understanding lung cancer metastatization to the lung. Therefore, besides discussing evidence focused on lung cancer metastasis, we will also review studies on the main mechanisms regarding lung metastasis originating from different primary tumors.

## 2 Dissemination and (pre)-metastatic niches

The first hypothesis suggesting that metastatic dissemination was not a “matter of chance”, was postulated in 1889 by Stephen Paget who noted in 735 cases of advanced breast cancer that not all organs were equally apt to receive “particles” from the primary tumor and develop metastasis (10).

Starting from this first evidence, Ewing and colleagues tried to explain the link between metastatic dissemination and the mechanical dynamics of hematogenous flow (11). Then, other studies have been conducted in the field concluding that certain organs were generally more susceptible to metastases than others (12). Finally, the now well-known “seed and soil” theory was put forward: tumor cells (“the seeds”) require an appropriate local microenvironment (“the soil”) to effectively grow as metastatic lesions.

Several efforts were then made to address the many open questions about how the primary tumor interacts with distant organs during the metastatic process (13). In this regard, a seminal work by Kaplan et al. in 2005 introduced the concept of “pre-metastatic niche” (PMN). This work demonstrated for the first time that the early recruitment of bone marrow-derived cells (BMDC) to the lungs caused microenvironmental changes and the creation of a pro-metastatic environment *before* the homing of circulating tumor cells (CTC) (14). These findings support the concept that primary tumors can shape the microenvironment of distant organs before tumor cell colonization. Although similar to Paget’s and Ewing’s theories, the concept of the PMN is more complex since it postulates the potential of some factors derived from primary tumors to precondition specific organs, making them suitable sites for metastases. Indeed, it’s now clear that primary tumors actively prime stromal cells at distant organs to generate a supportive environment for the recruitment, implantation, survival, and outgrowth of tumor cells (14–17).

In a general view, the establishment of the PMN is a multifaceted and multi-step process characterized by the requirement of fine-tuned communication between different sites and microenvironment players (11, 15). As recently reviewed by Peinado et al. (11), the establishment of PMN occurs through sequential steps: first, the blood vessels of the future site of metastasis lose their integrity in a phenomenon called “vascular leakage”, causing increased permeability and allowing the entrance of cells and macromolecules, which usually cannot otherwise penetrate the endothelial barrier. Next, other local stromal cells undergo a wide range of alterations; among them, fibroblasts play a central role through the deposition and remodeling of the extracellular matrix (ECM) or the secretion of molecules for immune cell recruitment and/or modulation (18, 19). The ensuing vascular and stroma rearrangement induces the recruitment of non-resident cells, mostly BMDC, that in combination with other deregulated immune cells generate a supportive environment for disseminated tumor cells and for subsequent metastasis outgrowth (11, 15, 20).

Several studies have described molecular and cellular effectors of this “systemic effect” connecting communication among different tissues (the site of the primary tumor, the bone marrow, and the site of metastasis) and proving the central role of tumor-derived extracellular vesicles (EVs) in PMN establishment (11). Since the role of other soluble factors, such as cytokines and growth factors, in the formation of the PMN was already well described (21), in this review we will focus on EVs, with a particular interest in the metastatic lung cancer setting.

## 3 Extracellular vesicles

Originally described simply as “waste operators”, EVs have recently gained attention for their role as key players in cell-cell communication (22). EVs are a large and heterogeneous group of cell-derived membranous vesicles secreted by almost all cell types (23, 24). Their family comprises several vesicles different in features (mainly in their size) and biogenesis. Despite the controversial and still evolving classification, EVs can be broadly subdivided into small EVs (sEV, also known as exosomes) derived from multivesicular bodies of late endosomes (~50–150 nm in diameter), and microvesicles (MVs or ectosomes) which originate through extracellular membrane budding (from ~100 nm up to 1 µm in diameter) (22, 25).

The bioactive cargo of EV comprises mostly transmembrane proteins, lipids, and nucleic acids as DNA and RNA (mRNAs, long non-coding RNAs, and miRNAs) (26–28). Although it is still unclear how proteins and nucleic acids are actively sorted or packaged into EVs, it is well established that the cargo of EVs is systematically integrated into the EVs through a strictly controlled process. In this regard, it has been proposed the involvement of the proteins of the Endosomal Sorting Complex Required for Transport (ESCRT) both in EV biogenesis and in particular during multivesicular bodies (MVBs) and intra-luminal vesicles (ILVs) formation and release (29–31). Indeed, the ESCRT-associated proteins Alix and TSG101 were found inside EVs, corroborating the role of ESCR complexes during cargo packaging. Even though the entire process is not yet fully understood, there is consensus about the requirement for some post-transcriptional modifications such as ubiquitination, SUMOylation, ISGylation, Phosphorylation, and Glycosylation (reviewed in detail by Sushma Anand and colleagues (32)) during the selective incorporation of cargo components. Indeed, the ESCRT complexes were found to be actively involved in the incorporation of ubiquitinated proteins inside EVs (33, 34) and the aforementioned Alix appears to play an active role in this process, sorting both proteins and miRNAs inside EVs (35, 36).

The content of EVs is cell-specific and reflects the physiological status of the cell of origin (37). For this reason, the presence of specific markers on the surface of EVs can be used to identify the cell type responsible for their release. For example, the tumor antigen 5T4 was detected in EVs from prostate cancer cell cultures and patients’ urinary-EV, but not in urinary EVs from healthy donors highlighting their specific tumor origin (38). In this scenario multiplex bead-based platforms allow simultaneous detection of several markers on EVs surface linked to the cell of origin. For instance, it has been demonstrated that NK cells-derived EVs are characterized by CD2, CD8, and CD56 markers whereas platelet-derived EVs lack CD2 and CD8 but are enriched in platelet markers such as CD41b, CD42a, and CD61 (39).

EVs have been successfully purified from many body fluids such as blood, urine, pleural effusions, ascites, and bronchoalveolar fluid (40), so they represent an interesting and non-invasive source of biomarkers for specific disease detection

The EV’s cargo is protected from enzymatic degradation by the phospholipidic bilayer allowing delivery of their content to recipient cells without any alteration (41). Once released, EVs can modify the physiological state of near or distant recipient cells by adhesion, fusion, and transfer of cargo components. The process of EV uptake by recipient cells is not yet fully understood but it occurs mainly in two possible ways: internalization by endo- and/or phagocytosis or by direct fusion with the membrane of the target cell. In both cases, the EV content is released into the cytosol of recipient cells. As a potential third mechanism of bioactive action, EVs can activate downstream signaling via receptor-ligand interaction on target cells (42). Several EV components (tetraspanins, integrins, or adhesion molecules) have been described as mediators of the binding between EVs and recipient cells (43). Indeed, the presence of specific integrins on the surface of cancer cell-derived EVs has been highlighted as responsible for the EV-cell interactions in different organs also during the PMN formation (28). Moreover, a specific pattern of tetraspanins on EVs has been shown to drive EV uptake by selected target cells, as indicated for example by the requirement of Tspan8 for the interaction between EVs and endothelial cells (44).

Following EVs uptake, their biological active cargo is released intracellularly to regulate in an autocrine and/or paracrine fashion multiple cellular processes including cell proliferation, survival, and potentially even transformation (43). Indeed, due to their ability to ‘deliver’ messages between different cells, EVs have been linked to all stages of cancer development, progression, and particularly to metastasis formation (45). Tumor-derived EVs indeed regulate different features of malignant cells such as proliferation (46), EMT (47), abnormal apoptosis (48), and metastatic spread (49).

### 3.1 Extracellular vesicles and (pre)-metastatic niche formation

As mentioned before, metastasis generation is a multistep process, beginning from the dissemination of primary tumor cells to the final colonization of distant organs. This process requires the acquisition of different properties and overcoming several hurdles making the overall frequency of metastasis a relatively rare event compared to the number of cells that leave the primary tumor. Firstly, by losing cell-cell contact and adhesion to the surrounding extracellular matrix (50) cancer cells start to invade neighboring tissues (51). Then, disseminating cancer cells enter the blood or lymphatic vessels and travel into the circulation where they must withstand several unfavorable conditions such as immune surveillance and anoikis (52). Cells able to survive in the circulation must finally extravasate through the endothelium and colonize the foreign parenchyma of distant organs to form metastasis (53, 54).

Primary tumor-derived EVs are supposed to be involved in all the steps of tumor dissemination and metastasis (55). Indeed, tumor derived-EVs sustain the metastatic cascade acting as mediators of intracellular communication between the tumor and the microenvironment. The first evidence of the involvement of tumor-derived-EVs in PMN formation was reported by Jung et colleagues in 2009, demonstrating that pancreatic cancer-associated exosomes actively participate in the establishment of lymphatic and lung PMNs in rodents (56). Using models of pancreatic adenocarcinoma, the authors demonstrated that highly metastatic tumor cells expressing CD44v were able to produce a soluble matrix which in cooperation with tumor-derived EVs altered stromal features of pre-metastatic organs. Indeed, concomitant *in vivo* administration of matrix+EVs from highly metastatic cells caused an increase in lymphocyte infiltration and endothelial cell activation that concurred in enhancing the metastatic potential of poorly metastatic cells (CD44v KO).

After that, several studies highlighted the involvement of EVs and their cargo in this process (57); however, only a few studies focused on the role of EVs from lung cancer cells in the formation of lung-PMN, which represents the first site of lung metastatization (7, 58–60). Therefore, we will highlight the studies reporting on lung metastasis from other primary tumors (breast, colorectal cancer, and melanoma) to describe the interplay between tumor-derived EVs and lung pre-metastatic microenvironment.

#### 3.1.1 EV-mediated modulation of stromal cells

One of the earliest events in metastatic niche formation is the increase of vascular permeability to facilitate cell extravasation. The modulation of the endothelial compartment during PMN formation was already demonstrated in the seminal paper by Jung et al. in rat pancreatic adenocarcinoma models: the authors reported an increased expression of genes involved in endothelial regulation (uPAR, VEGFR1, VEGFR2) induced by EVs secreted by highly metastatic cancer cells. Notably, alterations of the endothelial wall at the pre-metastatic lungs (hyper-permeability, altered morphology of vascular endothelium, and breakdown of the vascular basement membrane) were also noticed in other animal experimental models (61).

Vessel leakiness at pre-metastatic sites caused by primary tumor-secreted factors was reported in several papers. In this regard, Peinado and colleagues first demonstrated the involvement of melanoma derived-EVs in the induction of endothelial leakiness at the pre-metastatic sites (lung). Indeed, the authors showed that endothelial permeability, assessed *in vivo* using fluorescent dextran, was markedly increased after treatment with EVs from metastatic cell lines compared with treatment with EVs from non-metastatic cell lines. The authors suggested that TNF-α, up-regulated in lung tissue soon after EV administration, could play a pivotal role in this process (26). Then, other studies investigated the interplay between cancer EVs and endothelial cells. Indeed it has been reported that EVs derived from lung cancer or breast cancer cells carrying miR-23a and miR-105 respectively, increased vascular permeability and facilitated cancer cell colonization by targeting the tight junction protein ZO-1 (62, 63). Moreover, cancer-derived EVs can activate the endothelium and promote lung PMN formation through the upregulation of MMP2, MMP9, and VEGFR1 (64). Importantly, the perturbation of the lung endothelial wall by tumor-EVs was also demonstrated in a model of hepatocellular carcinoma (HCC), where tumor-EVs carrying Nidogen 1 (NID1) enhanced angiogenesis and pulmonary endothelial permeability (65). In the same study, the authors also highlighted that NID1^+^-EVs activate fibroblasts that in turn facilitated lung colonization by secreting the tumor necrosis factor receptor 1 (TNFR1). Another study reported the role of pancreatic cancer Tspan8-EVs in modulating both lung endothelial cells and fibroblasts to favor metastatization (66). Interestingly, miR-122 cargoed by breast cancer-EVs can impair the glucose consumption of lung fibroblasts, resulting in increased microenvironmental availability of glucose that supports metastatic cell proliferation (67). Interestingly, miR-122 was found to be selectively incorporated and enriched in NSCLC cell lines-EVs (68), suggesting a possible role of these EVs also in lung cancer metastasis.

#### 3.1.2 Immunoregulatory activity of EVs

The lung metastatic niche is a complex microenvironment comprising several stromal and immune cells, which include T cells, monocytes, neutrophils, NK cells, and macrophages. Immune cells could potentially target tumor cells and prevent their growth and dissemination: cancer cell-derived EVs in turn contribute to the escape from the anti-tumor activity of immune cells.

One relevant example is represented by lung cancer-derived EVs carrying programmed death ligand 1 (PD-L1) on their surface. The binding of PD-L1 to PD-1 expressed on cytotoxic T cells inactivates their function promoting tumor growth and metastasis (69). Moreover, the presence of PD-L1 on lung cancer-derived EVs suppresses the differentiation and maturation of dendritic cells (DCs) in mouse models. Exosomes can induce the expansion of T regulatory cells and this effect can be abolished by blocking PD-L1 (70). In another paper, circulating EVs isolated from the plasma of lung cancer patients were shown to display high levels of EGFR on their surface able to induce tolerogenic DCs that promote tumor-specific T regulatory cells (71). Furthermore, lung cancer cells under hypoxic conditions release microvesicles with high amounts of TGF-β and miR-23 able to block the cytotoxic activity of NK cells *in vitro* and *in vivo* (72). In addition, exosomal miR-21/29a released by lung cancer cells activates TLR7 and TLR8 on macrophages triggering the NF-kB pathway and release of inflammatory cytokines that sustain tumor growth and metastasis (73). A study conducted using both mouse models and cell lines showed that primary tumor-derived exosomal RNAs mediate the activation of TLR3 in lung epithelial cells inducing chemokines release that in turn recruit neutrophils from bone marrow to the PMN site (59).

One of the main limitations of these studies is the frequent use of EVs from cancer cell lines, which are normally tested at much higher concentrations compared to the relative abundance of tumor EVs in the blood of cancer patients: therefore it should be stressed the need to scale down the concentration of cancer cell line-derived EVs to properly mimic the role of tumor EVs in organ intercommunication (like pre-metastatic niche formation) occurring in cancer patients.

EVs are released also as a consequence of the crosstalk between the tumor and its microenvironment and could influence cells at distant organs. Therefore, it could be very relevant to evaluate the cellular origin of EVs from the primary tumor and its microenvironment; the isolation of EVs from fresh tissue samples and their characterization using a multiplexing approach will be helpful in identifying EVs originating from different cell types. Moreover, based on antibodies selective for surface markers, EVs released from different cells of origin could also be sorted and used to better understand the mechanisms and the major players involved in the first steps of lung metastatization.

## 4 Role of EVs in cancer cell-tumor microenvironment crosstalk at primary tumor sites

**Figure 1:**
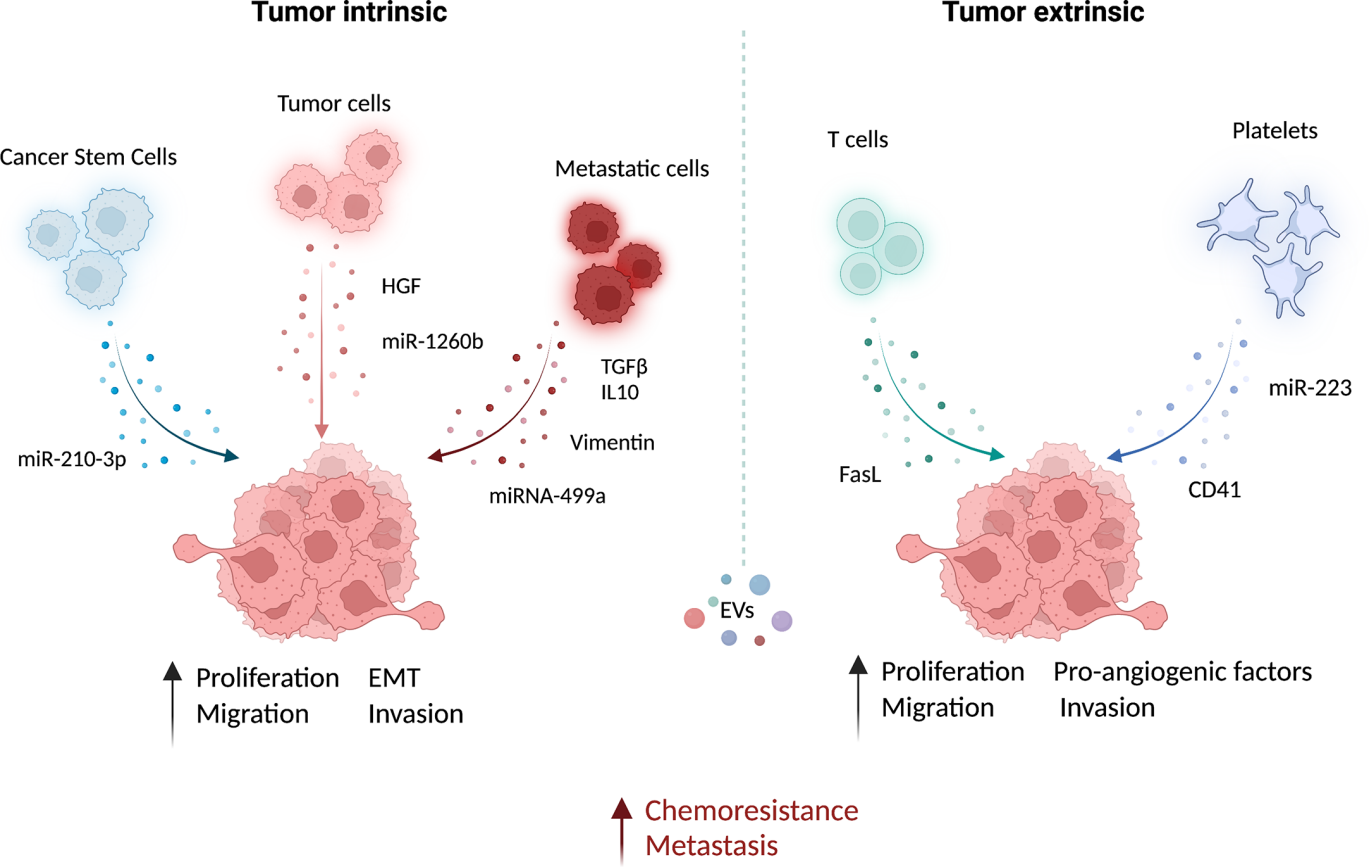
Functional role of EVs in the microenvironment of lung cancer. Extracellular vesicles (EV) from different cell types can contribute actively to prompt tumor growth and metastatic dissemination. The EV-mediated delivery of bioactive molecules, such as miRNAs and proteins, from different cell types to cancer cells has been linked to the induction of pro-metastatic features and chemoresistance. This intercellular communication within the tumor may occur both via intrinsic (between cancer cells) and extrinsic (e.g. between immune cells and cancer cells) EV exchange. EVs from lung cancer-stem-like cells (CSC) increase migration and invasion of lung cancer cells (83). Similarly, EVs from lung cancer tumor cells carrying the hepatocyte growth factor (HGF) (79) and miR-1260b (77) can increase cancer cell epithelial-mesenchymal transition (EMT) and migration, respectively. Moreover, proliferation, EMT, and migration capabilities of non-metastatic lung cancer cells and non-tumoral lung epithelial cells can be increased by metastatic cells derived EV enriched in TGF- and IL-10 (75), vimentin (47) and miRNA-499a-5p (76). The growth and metastatic potential of tumor cells can be also modulated by non-tumoral-EVs. EV from CD8 T cells can increase the cell metastatic behavior by FasL (82). Platelets also play a fundamental role in promoting cell invasion and angiogenesis by releasing EV-containing miR-223 (80) and CD41 (81), respectively. This image was created with BioRender.

EVs are involved in the acquisition of mesenchymal and more aggressive phenotypes by lung cancer cells (**Table 1**, **Figure 1**). In this context a prominent role is played by cells endowed with increased tumor-forming potential: the existence of a subset of cancer stem-like cells (CSC) able to sustain primary tumors and initiate distant metastasis has been extensively demonstrated in both hematological and solid tumors, including lung cancer in which CSC have been identified by the expression of CD133 marker by different groups (84, 85). Expression of CSC markers in primary tumors has been shown to correlate with worst prognosis and metastasis occurrence. Recently, some evidence also highlights the importance of CSC-derived EVs in mediating aggressive phenotype of tumors or in corrupting surrounding and distant stroma cells to promote tumor progression (86). Indeed, in different tumor types, it has been demonstrated that CSC- EV cargo can induce non-tumor cells to gain stem-like features through the induction of EMT which in turn promotes chemotherapy resistance and metastasis formation (83, 87, 88). Different bioactive molecules carried in CSC-derived EVs have been shown to mediate such effects, among which stemness-related proteins or activators of stem-related signaling (89, 90) and RNA molecules (91).

**Table 1:** Extracellular vesicles as modulators of phenotype of primary tumor.

Tumor intrinsic			
EV’s Cargo	Origin	Function	Ref
miR-210	Cancer Stem Cells	Activation of FGFRL1 and metastasis	(74)
TGF-β/IL-10	Metastatic cancer cells	Increase of proliferation and migration	(75)
Vimentin	Metastatic cancer cells	Induction of EMT	(47)
miR-499-5p	Metastatic cancer cells	Induction of EMT, proliferation and migration	(76)
miR-1260b	Lung cancer cells	Modulation of sFRP1 and SMAD to increase invasiveness	(77)
Tetraspanin 8	Metastatic lung cancer cells	Promote invasiveness	(78)
HGF	Metastatic lung cancer cells	Promote migration and proliferation through c-Met	(79)
Tumor extrinsic			
miR-223	Platelets	Promote invasion	(80)
CD41	Platelets	Increase migration and cytokine release	(81)
FasL	CD8 T cells	Stimulate metastasis	(82)

In lung cancer, it has been demonstrated that CSC-derived exosomes can enhance the invasive and pro-metastatic properties of lung cancer cells by transferring miR-210-3p that binds and down-modulates fibroblast growth factor receptor-like 1 (FGFRL1), inducing a mesenchymal phenotype (74).

Interactions among different subsets of cancer cells can also be mediated by EVs: metastatic cell-derived EVs can stimulate the acquisition of aggressive behavior in recipient cells. Indeed, exosomes isolated from metastatic small-cell lung cancer cells increased cancer cell proliferation and migration compared to non-metastatic cells through the release of EV-derived TGF-β and IL-10 (75). Moreover, exosomes released by metastatic lung cancers can induce EMT in normal epithelial cells by transferring vimentin (47). miRNA-499a-5p, highly enriched in EVs from metastatic lung cancer cells, modulates the mTOR pathway in recipient lung cancer cells with concomitant induction of proliferation, EMT, and migration (76). The presence of miR-1260b in lung cancer-derived EVs promotes the invasive capacity of lung tumor cells through the modulation of the sFRP1 and SMAD4 pathway (77). Additionally, Tetraspanin 8 on the surface of EVs stimulates the invasiveness of both human and mouse lung cancer cells in *in vitro* studies (78). Interestingly, EVs can also be able to promote proliferation and migration of lung cancer cells by transferring HGF and concomitant activation of c-Met (79). Tumoral-derived exosomes detected in the pleura exudates carry enzymes for leukotriene (LT) biosynthesis that can act on lung cancer cells promoting their migration and proliferation (92).

Exosomes from cancer cells can be exploited as a communication tool with local and distant normal cells to generate a micro-environment suitable for their proliferation/invasion. For instance, lung cancer cell-derived exosomes were able to induce a pro-inflammatory phenotype in mesenchymal stem cells that in turn support tumor growth (93).

Similarly, exosomes from immune/stroma cells can in turn contribute to the induction of aggressive properties in recipient tumor cells. Microvesicles released by activated platelets in patients with lung cancer are also involved in metastatic outgrowth. Indeed, these vesicles are enriched in miR-223 compared to healthy donors and this miRNA promotes cell invasion through the inhibition of erythrocyte membrane protein band 4.1-like 3 (EPB41L3) (80). Another study revealed that microvesicles from tumor-educated platelets enhance the migration of lung cancer cell lines and increase the release of angiogenic factors such as VEGF, MMP-9, and IL-8. The authors demonstrated also that the presence of integrin CD41 inside EVs can enhance the metastatic potential of murine lung cancer cells (81). In addition, activated CD8 T cells release exosomes expressing FasL that can modulate MMP-9 expression in cancer cells and consequently lung metastasis (82).

## 5 The journey of circulating tumor cells to the activated PMN

Primary tumors constantly shed millions of cells per day into the bloodstream, an event that also occurs at the early stages of the disease (94). Circulating tumor cells (CTC) represent the seed of metastasis and their targeting in the circulation, or once landed at the PMN, represents a challenging but crucial requirement to counteract metastasis formation. Despite the very low number of CTCs that can be detected in the blood of patients at specific time points (1 CTC:10^7^ leukocytes), these cells are characterized by extreme heterogeneity, detected at genomic, transcriptomic, and functional levels which greatly limits their in-depth study and characterization (95).

Considering the great number of CTCs released by primary tumors during development and expansion, the efficiency of metastatic seeding is extremely rare, with an estimated success rate of 0,001% (96). This observation subtends that only very few CTCs can survive in the circulation, reach PMN and initiate metastasis. Rising evidence is emerging highlighting that the metastatic potential of CTCs is largely confined to the cell subset endowed with stemness and mesenchymal traits that could succeed to carry out all the steps of the metastatic cascade (97–99). Recent technological advances in single-cell sequencing strategies have allowed a better understanding of CTC heterogeneity in different tumor types and confirmed, in preclinical models and patient samples, the activation of stemness programs in CTCs, strongly supporting the existence of a rare subpopulation of stem-like cancer cells guiding tumor spread and metastasis initiation (100–102).

CTCs can enter the circulation as single cells or, occasionally, as clusters of tumor cells alone or associated with immune cells or stroma cells, such as neutrophils or cancer-associated fibroblasts (103). Clusters of tumor cells, held together by cell-cell junctions (104) or cytoskeletal proteins (105) can be shed from primary tumors; alternatively, clusters can be formed in the circulation by single CTC aggregation through homophilic interactions (106). Several studies have demonstrated in different tumor types that clusters and in particular heterotypic clusters, comprising CTCs and immune/stroma cells, possess the highest metastatic ability (104) and their detection in patients’ blood predicts worst outcome (104, 107–109).

Neutrophils are the most common and ‘dangerous’ travel companions of CTCs. It has been demonstrated that neutrophils can escort circulating tumor cells and support their proliferation by enabling cell cycle progression: as a result, once landed at the PMN, active CTCs have a higher chance to initiate metastasis (109). Besides neutrophils, other immune cells and stroma cells can travel in circulation with CTC. For instance, platelets bound to CTCs can have multiple protective effects, by preventing immune cells’ recognition and attack of CTCs and by providing factors able to support CTCs’ survival in unfavorable conditions or conferring malignant traits, such as acquisition of CSC phenotype, invasiveness, and drug resistance (110). Macrophages that bind to CTCs play a crucial role in tumor cell intra and extravasation steps, supporting CTC seeding and survival at distant sites (111). Finally, Duda et al. demonstrated that also cancer-associated fibroblasts can travel into heterotypic CTC cluster that spontaneously spread from the primary tumor and this interaction greatly help CTCs to establish distant metastasis (112).

Once landed at the PMN, CTC can interact with (pre)activated endothelial cells and immune cells that can facilitate their entrapment and survival. In particular, it has been demonstrated that neutrophil extracellular traps (NETs) can capture CTC through a β1-integrin-mediated mechanism and this interaction can sustain CTC potential in metastasis development (113).

Even though no direct evidence has been reported regarding the role of EVs in dictating the metastatic behavior of CTCs, it is conceivable that EVs released by both immune/stroma cells and cancer cells can mediate the process leading to CTCs escape from the primary tumor, survival in circulation and seeding at distant sites. Indeed, as discussed in the previous paragraphs, EVs released from stroma and immune cells may participate in the process of cancer cell intravasation and formation of the metastatic clusters as well as could mediate the activation of stemness/survival/proliferative pathways in CTCs. Finally, CTCs attracted to EVs-primed PMN can find the most favorable soil to initiate metastasis.

## 6 Dynamics at distant sites: metastatic niche formation

**Figure 2:**
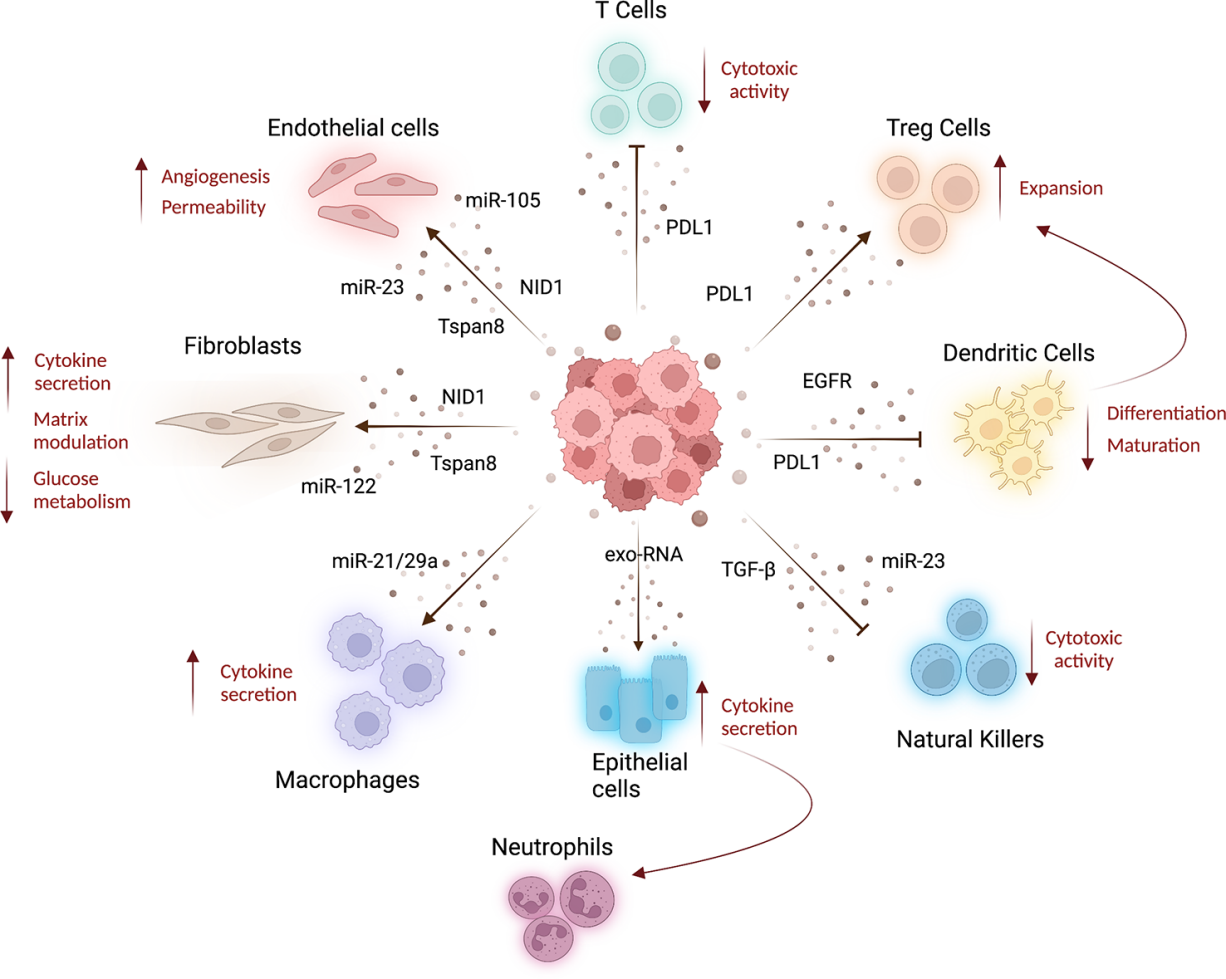
Systemic effects of cancer derived-EVs. Lung cancer tumor-derived EVs released into the bloodstream can be taken up by several cell types affecting the features of stroma and immune cells at distant organs, resulting in cancer progression and metastatization. Indeed, tumoral EVs released in circulation can interact with different cell types inducing stromal alterations. For example, tumor-EVs can transfer miRNAs to increase endothelial wall destabilization and permeability (61, 62), whereas Tspan8-EV induces angiogenesis (44). Moreover, Tspan8-EV (66) and tumoral EVs carrying nidogen 1 (NID1) (65) can facilitate lung-premetastatic niche formation and lung colonization, acting both on endothelial cells and fibroblast and inducing angiogenesis, endothelial permeability, and cytokines released by fibroblasts. Fibroblast metabolism is also affected by miR-122, enriched in tumoral EVs, which induces low glucose consumption (67). Tumor-EVs can modulate T-cell compartment, decreasing their cytotoxic activity and inducing the expansion of T-reg cells (by PDL1-EV), resulting in immunosuppression (69, 70). PDL1-EVs can also modulate dendritic cell activity decreasing their differentiation and maturation (70), whereas EGFR-enriched EVs prompt a tolerogenic phenotype (71). To increase immunosuppressive environment, tumor EVs can also deliver TGF-β and miR-23 to natural killer cells blocking their cytotoxic activity (72). The activation of pro-inflammatory immune cells is also a feature of pre-metastatic niche formation. Indeed, tumor-EV can indirectly increase cytokine production in lung epithelial cells that in turn recruit neutrophils to the lung (59). Moreover, EV-miR-21/29a released by lung cancer cells increases macrophage cytokines production to sustain metastasis formation (73). This image was created with BioRender.

After the arrival at metastatic sites, disseminating tumor cells could be eliminated by immune cells or enter a state of dormancy by exiting proliferative cell cycle (54). Remodeling of lung stromal cells is a key step to generate a fully competent metastatic niche before or also after the arrival of CTCs (**Figure 2**). The recruitment of immune cells with an immunosuppressive and pro-metastatic phenotype appears, however, crucial for the development of metastasis. In a murine model of lung carcinoma, tumor cells activated through TLR7 are able to recruit myeloid-derived suppressor cells (MDSCs) via the release of cytokines such as CCL2 and GM-CSF (114). The expansion of the MDSC pool with an immunosuppressive phenotype in the lungs leads in turn to metastatic progression.

Neutrophils emerge as one of the most important immune cells that could control the metastatic process in lung. The recruitment of neutrophils by lung epithelial cells activated by tumor-derived exosomal RNAs-TLR3 binding was recently shown to be an important step in PMN formation by supporting the growth of lung cancer cells (59). Interestingly, it has been also demonstrated that neutrophils reactivate dormant cancer cells by switching their polarization status. Indeed, stress-activated PMN-MDSCs release S100A8/S100A9 which can support the reactivation of dormant tumor cells (115).

In some cases, dormant cells can activate bone-marrow-derived endothelial progenitor cells by promoting an angiogenic switch that allows angiogenesis-mediated progression of micro-metastasis to overt metastasis (116). Since the formation of the metastatic niche is a process that involves several cell types, further studies are needed to elucidate the cross-talk between EVs, stroma, and immune cells at distant organs. Little is known about the role of EVs derived from other cell types at secondary organs in modulating the environment for metastatic outgrowth. It could be also possible that EVs control tumor cell dormancy although this process is not yet fully understood. An interesting work showed that the presence of miR-210 and miR-193 inside EVs released by hypoxic bone marrow-derived cells (BMDC) can increase lung cancer cells invasion and EMT through the activation of STAT3 pathway (117). Recruited BMDC contribute to the formation of liver metastasis from lung cancer by releasing EVs enriched in miR-92a (118). In the brain, EVs shed by endothelial cells exert a protective effect on the survival of SCLC cells through the up-regulation of S100A16 (119).

Even though it appears clear that EVs derived from cells at the metastatic niche could regulate the fate of disseminating cells, it is hard to establish the exact origin of these EVs. Moreover, the majority of published studies were focused on the ability of EVs to induce an aggressive phenotype in lung cancer cells while very little is known regarding EVs-induced mechanisms that regulate the transition from DTC to full metastasis.

### 6.1 Dormancy

Along the metastatic cascade, tumor cells that leave the primary tumor and reach distant organs (disseminated tumor cells, DTCs) can remain in a quiescent state for a variable amount of time: this process is often referred to as dormancy and has important clinical and biological implications (54). Coupled with the evidence that dissemination could be a very early event in tumorigenesis (120, 121), the observation that cancer cells can possibly persist as disseminated tumor cells even for decades has in fact spurred considerable efforts towards the understanding of biological mechanisms regulating dormancy and awakening.

From the clinical perspective, dormancy is believed to be the underlying mechanism subtending late relapses after a prolonged ‘tumor-free’ period following primary tumor removal in particular in some tumor types, including ER+ breast cancer, melanoma, prostate, and renal cancer. In this context, the elucidation of pathways involved in the maintenance of tumor dormancy and, most importantly, in the reactivation of dormant cells (awakening) is crucial to implement optimal follow-up strategies (122, 123). Furthermore, the potential of ‘dormancy-inducing’ therapies to block or convert minimal residual disease at the level of (few) DTCs is also being considered: as a paradigm of this concept the epigenetic regulation of dormancy-inducing nuclear receptor NR2F1 with *all-trans* retinoic acid and azacytidine (124) or with a specific agonist (125) has been shown to induce prolonged dormancy and reduce metastatic outgrowth in head and neck squamous cell carcinoma (HNSCC).

Most studies on quiescence have concentrated on tumor types with clinical evidence of very late relapses (e.g. breast cancer, melanoma) or with availability of very informative experimental models (HNSCC). In many cases, the lung was used as the primary metastatic site mimicking the clinical setting. As explained above these studies present evidence that could be relevant for primary lung cancer due to its propensity for local metastatic dissemination. Interestingly similar transcriptional programs are activated in quiescent cancer cells from lung and colorectal cancer highlighting the potential existence of general programs regulating quiescence in different settings with particular relevance for pathways controlling stemness and EMT together with signatures related to TGF-β signaling (126).

Dormancy of cancer cells is regulated both at the cell’s intrinsic and extrinsic levels and the contribution of the microenvironment is increasingly recognized as crucial in different phases of induction, maintenance, and exit from dormancy (127). Interestingly, interactions within the primary tumors have been shown to regulate phenotype and fate of dormant disseminated tumor cells through priming via a TGF-mediated mechanism (128), hypoxia (129), or even in relation to the time of dissemination from the primary tumor with specific properties identified in early disseminating cancer cells (eDCC) (121, 130).

At the cell-intrinsic level several pathways have been identified as being central for the dormant phenotype in different settings including the p38/ERK signaling ratio (131), CXCR4 activated Src-dependent signaling (132), endoplasmic reticulum stress (133), VCAM1 (134) or BMP-dependent signaling (135). Extracellular signals that often stimulate or converge on the activation of the same pathways can be the result of interactions with the extracellular matrix, stromal cells, and hypoxic or generally inhospitable microenvironments that can activate stress responses (136). Importantly, several traits of dormant cells are also similar to those of (metastatic) cancer stem cells implying potential overlap of phenotypes and relying on similar niches, arguing therefore for the importance of a unified view of different aspects of the metastatic cascade (137).

Extensive descriptions of the dormancy state have been provided elsewhere and are beyond the scope of this review (127). We will briefly summarize the available evidence on the regulation of dormancy by microenvironmental cues and the potential role of EVs in this mechanism. While several secreted factors have indeed been associated with dormancy the contribution of EVs is also beginning to be unraveled.

### 6.2 Regulation of dormancy by microenvironmental cues and EVs

Dormancy appears to be tightly regulated by interactions within the microenvironment (138). Disseminated cancer cells that reach distant organs often have to adapt to foreign sites and exploit existing niches. Modifications of the microenvironment that can induce pre-metastatic niches discussed previously have therefore also potential implications for the behavior of dormant cells (11).

As an example, dormant breast cancer cells often reside at the microvascular niches where dormancy is enforced through endothelial cell-derived thrombospondin (TSP1). In sprouting neo-angiogenesis however, this effect is reversed via TGFβ and periostin-mediated signaling resulting in an accelerated outgrowth of cancer cells (139). The perivascular niche has also been shown to provide clues to favor chemoresistance of DTCs via endothelial-derived von Willebrand factor (vWF) and VCAM1: disruption of niche interactions through integrin inhibition resulted in sensitization of DTCs to conventional therapy also providing intriguing evidence against a fully quiescent phenotype of DTCs (140). Although dissection of the potential role of EVs in these interactions was not provided it is interesting to note that endothelial cells are important producers of exosomes and that both vWF factor and VCAM1 have been described as part of the endothelial-derived exosome cargo (141).

In the context of lung cancer, quiescence has been investigated more broadly with respect to the effects of therapy-induced dormancy and relapses, which have been recently reviewed (142). It is important to note that there are however important similarities between the different phenotypes and similar strategies could be investigated to target disseminated tumor cells and drug-tolerant persister cells (143). Interestingly some effects of chemotherapy could also paradoxically alter the microenvironment towards a tumor (or metastasis) promoting state (144). We recently described that cisplatin can induce bone marrow expansion of CCR2^+^CXCR4^+^Ly6C^high^ inflammatory monocytes and an increase in lung levels of stromal SDF-1, the CXCR4 ligand. Recruitment of inflammatory monocytes in the lungs generates prometastatic niches for CD133+/CXCR4+ metastasis-initiating cells which can be prevented by a CXCR4 inhibitor (145). In the lungs, the effects of cisplatin are mediated through the activation of endothelial cells and disruption of α-SMA endothelial layers highlighting once more the relevance of vascular niches. Dormancy and reactivation of lung cancer cells following cisplatin treatment has also been shown to be dependent on a Sox2/Nanog regulated mechanism relying on sequential *cis* and *trans* ephrin type-B receptor 1 (EphB1)-mediated signaling (146). In particular, in the dormant state, the activity of EphB1 is ligand-independent and this could provide intriguing speculations regarding potential interactions within the tumor microenvironment since it has been described that exosomes could mediate cell-contact independent ephrin signaling to regulate different mechanisms including axon guidance and tumor angiogenesis (147, 148). Increased levels of regulator of G protein signaling 2 (RGS2) activated by chemotherapy-induced-ER stress have been identified in models of slow cycling/dormant cancer cells from non-small cell lung cancer cell lines and Patient-Derived Xenografts (149). RGS2 activity disrupts ATF4-mediated translational control and endows surviving cells with dormancy features and survival potential. Reversal of this mechanism through RGS2 antagonism sensitized cells to chemotherapy-induced apoptosis.

Interactions with the extracellular matrix (ECM) are crucial determinants of cellular behavior and regulate multiple biological programs including cell fate/determination, proliferation, and migration (150). ECM signals are active at biochemical, biophysical, and biomechanical levels and have profound implications for the generation of functional niches for both normal and cancer stem cells and consequently also for DTCs.

The recently identified presence of matrix-bound nanovesicles provides a potential new layer of complexity for the investigation of interactions within the ECM (151). Alterations of ECM dynamics have been shown to play crucial roles in reactivation of dormant cells (6). In a seminal observation, a lung fibrotic environment enriched in collagen-1 induced reactivation of dormant breast cancer cells through β1integrin- mediated activation of *Src* and focal adhesion kinase (152). The regulation of cytoskeleton reorganization appears to be a crucial aspect of the transition from dormancy to proliferation since the targeting of the reorganization can inhibit metastatic outgrowth *in vivo* (153). Intrinsic regulation of ECM composition by tumor cells is also an important factor as demonstrated by the observations that dormant cancer cells produce instead a type III collagen-enriched niche required for sustained dormancy (154). Post-translational modifications play an additional important role as exemplified by the observation that collagen glycosylation regulates stemness phenotype and proliferation in lung cancer (155).

Recently interesting evidence has also appeared regarding the relevance of microenvironment changes associated with aging (156). The physiological processes associated with aging have in fact an important impact on different tissues and may modify local microenvironments permissive for tumor growth or for proliferation of dormant cells. According to this hypothesis, a non-canonical Wnt antagonist produced by aged lung fibroblasts (sFRP1) was found to induce the awakening of melanoma cells in the lungs (157). Interestingly a similar secretome (sFRP2 and WNT5) from aged skin fibroblasts induced instead slow growing but disseminating melanomas (158) potentially highlighting tissue and/or time-specific mechanisms. sFRP2 was also shown to be relevant for alveolar type 1 cells mediated dormancy of breast cancer cells associated with formation of fibronectin fibrils (159). Together with the previously discussed evidence that stress-activated neutrophils can induce reactivation of dormant lung cancer cells (115), these data suggest that many host-related factors, including aging and stress, could be at play to determine the fate of dormant cells. Interestingly the activation of the glucocorticoid receptor has recently been shown to be associated with the induction of a reversible dormant state, associated with resistance to anticancer drugs suggesting caution for use of glucocorticoids during cancer therapy and highlighting the complexity of the scenario (160).

Overall further research is warranted on many aspects of tumor-stroma interactions as determinants of metastatic outgrowth to fully understand the underlying mechanisms and devise novel therapeutic strategies targeting the (pre)metastatic niche.

## 7 Conclusions

Metastatic disease remains the first cause of lung cancer-related deaths. The complexity of the metastatic cascade steps and the intricate connections and cross-talk between cancer cells and the microenvironment constitute challenges for a deeper understating of advanced disease and the possibility to prevent/counteract it. It’s becoming clear that the generation of specialized microenvironments at distant sites, named the pre-metastatic niche (PMN), guides the propensity of primary cancer cells to colonize specific distant organs and dictates their success to initiate metastasis.

Primary tumor-secreted factors are able to prime stromal and immune cells at distant sites thus creating a permissive niche for circulating tumor cells landing and metastatic colonization. Among secreted factors, extracellular vesicles have been recently recognized as central mediators for PMN formation. EVs cargo is composed of a plethora of bioactive molecules that impact all different steps of the metastatic cascade. EVs released by primary tumors can induce vascular leakiness at distant sites, activation of fibroblasts, and recruitment of BMDC, events that all together concur in PMN formation. On the other hand, at primary tumor sites EVs released by both cancer and stromal/immune cells can impact on the acquisition of invasiveness and stem-like cancer phenotypes and can favor tumor cells’ intravasation and dissemination.

Circulating tumor cells can be directed by stimuli within the primary tumor to reach distant organs, where they can interact with EVs-primed stromal/immune cells which favor their extravasation and govern cancer cells’ dormancy/awakening, dictating metastatic success.

Here we have reviewed available evidence highlighting the involvement of EVs in all the different steps of metastasis formation in the context of lung cancer. Although the precise role of EVs has yet to be elucidated for a comprehensive view of the process, it is clear that they contribute to the successful development of PMNs and metastasis formation. Therefore, a deeper knowledge of EVs’ origin and cargo can shed new light on our understanding of metastatic disease and may allow the identification of novel targets to prevent lung cancer progression and metastasis formation.

## Author contributions

Writing—original draft preparation: FP, LR, OF, and GB. Writing—review and editing: FP, LR, OF, and GB. Funding acquisition: OF and GB. All authors have read and agreed to the published version of the manuscript. All authors contributed to the article and approved the submitted version.
